# Outcomes following repair of anomalous coronary artery from the pulmonary artery in infants: results from a procedure-based national database

**DOI:** 10.1136/openhrt-2015-000277

**Published:** 2015-09-04

**Authors:** Daniel Paul Fudulu, Dan Mihai Dorobantu, Mansour Taghavi Azar Sharabiani, Gianni Davide Angelini, Massimo Caputo, Andrew John Parry, Serban Constantin Stoica

**Affiliations:** 1 University Hospitals Bristol NHS Trust, Bristol, UK; 2 Imperial College, London, UK; 3 Rush University Medical Centre, Chicago, USA

**Keywords:** CONGENITAL HEART DISEASE, QUALITY OF CARE AND OUTCOMES

## Abstract

**Background:**

Anomalous coronary artery from the pulmonary artery (ACAPA) is a very rare congenital anomaly that often occurs during infancy. Patients can present in a critical condition.

**Methods:**

We analysed procedure-related data from a national audit database for the period 2000–2013.

**Results:**

A total of 120 patients <1 year had repair of isolated ACAPA using a coronary transfer or the tunnel (Takeuchi) operation. Seven patients (6.8%) required a mitral valve procedure at index and eight patients (7.8%) had a mitral valve repair/replacement during follow-up, including mitral reoperations. Follow-up data (>30 days) were available in 102 patients and the mean follow-up time was 4.7 years. The 30-day overall mortality was 1.9%, higher for neonates (16.7% vs 1%, p=0.1) and after postoperative extracorporeal membrane oxygenation (ECMO) (20% vs 1%, p=0.09). At 10 years the survival estimate is 95.1%, freedom from coronary and mitral reintervention being 95.9% and 91.2%, respectively. Use of postoperative ECMO was a risk factor for long-term mortality (p<0.001). Risk factors for coronary reintervention were age under 30 days (p=0.06) and the need for postoperative ECMO (p=0.02). Age under 30 days (p=0.002) was a risk factor for mitral reintervention.

**Conclusions:**

To our knowledge this is the largest series to date. These preliminary national results show that early outcomes are good and medium-term attrition acceptable. Poor outcomes are correlated with early presentation, also with the need for postoperative circulatory support.

**Key questions**

**What is already known about this subject?**

- Anomalous coronary from the pulmonary artery (ACAPA) is a rare anomaly which usually presents in infancy with ischaemic cardiomyopathy. This may result in heart failure and various degrees of mitral regurgitation. Current treatment options include establishment of a dual coronary artery system using typically direct coronary transfer from the aorta to the pulmonary artery. On small series, the early and long-term outcomes are excellent. Decreased left ventricular function and young age at operation are the most common known risk factors for poor outcomes. Following repair, most studies report an improvement in myocardial function followed by resolution in most cases of the functional mitral regurgitation. Timing of mitral valve repair/replacement remains a controversy; however, most authors agree about repairing structural defects at the time of ACAPA surgery while functional mitral regurgitation is not addressed.

**What does this study add?**

- This study reports early and medium-term outcomes following repair of ACAPA in the largest cohort to date.
- ACAPA repair is infrequent in individual centres due to the rarity of this condition, nevertheless the results are excellent.
- Coronary reinterventions are infrequent and they all occurred in the first year of follow-up.
- Age below 30 days was a risk factor for coronary and mitral reintervention while use of postoperative extracorporeal membrane oxygenation negatively influenced coronary reintervention and long-term survival.

**How might this impact on clinical practice**

- ACAPA repair is achieved with uniformly good outcomes.
- Monitoring for myocardial ischaemia is warranted during early and medium follow-up.

## Introduction

Anomalous origin of the coronary artery from the pulmonary artery (ACAPA) is a very rare anomaly which usually occurs in isolation. ACAPA can be classified in infant ACAPA and non-infant (adult) ACAPA. They have a different clinical presentation and mechanism of disease. In infancy, the vast majority of patients present with the anomalous left coronary artery from the pulmonary artery (ALCAPA) rather than the right coronary connection (ARCAPA).^1^ Severe forms of ARCAPA are usually associated with right coronary dominance.^1^ Some infants develop ischaemic cardiomyopathy, resulting in left ventricular (LV) failure and various degrees of mitral regurgitation. A small number of patients do survive until childhood or adulthood untreated, due to secondary coronary collateralisation.^2^

Both infant and adult ACAPA variants need urgent surgical repair. Reported short and long-term results vary, depending on centre experience, patient characteristics and technique used.^2–5^ The aim of this multicenter study is to report outcomes following the repair of infant anomalous coronary artery from the pulmonary artery using coronary transfer or the tunnel operation in consecutive patients treated nationally.

## Patients and methods

### The data set

The National Institute for Cardiovascular Outcomes Research (NICOR) collects validated key data on cardiac procedures from all the UK heart units with the aim of providing information on surgical and catheter-based procedures and outcomes. The data is stored in the Central Cardiac Audit Database (CCAD). NICOR's mechanism for congenital data capture, cleaning and validation is similar to other databases adopted for adult cardiac surgery.^6^ Using linkage with census records at the Office of National Statistics, the audit database publicly reports survival rates at 30 days and 1 year following the index procedure online (available at: https://nicor4.nicor.org.uk/). This data is periodically audited, both internally in each centre and also externally by NICOR. Linkage with survival registries of Northern Ireland and Scotland cannot be carried out consistently with their personal identification number, while a minority of patients either have errors in their social data or are foreign. This resulted in 15% of patients not having follow data beyond 30 days. Indications for each operation were given by the multidisciplinary teams at each centre. Additional diagnoses are as selected by the reporting clinicians and concordance between diagnoses and procedures performed are actively audited. The data quality index for key procedure fields is above 95%. The need for patient-level consent to participate in this retrospective study was waived by the NICOR Research Board. Diagnosis and procedure codes from the European Pediatric Cardiac Code Short List are used. A total of 19 (15.4%) patients were reported to have either LV failure or cardiomyopathy, but since there are no guidelines for reporting LV function to NICOR we believe that this figure is an underestimation. We did not use these data in the analysis.

#### Patient selection

**Figure 1:**
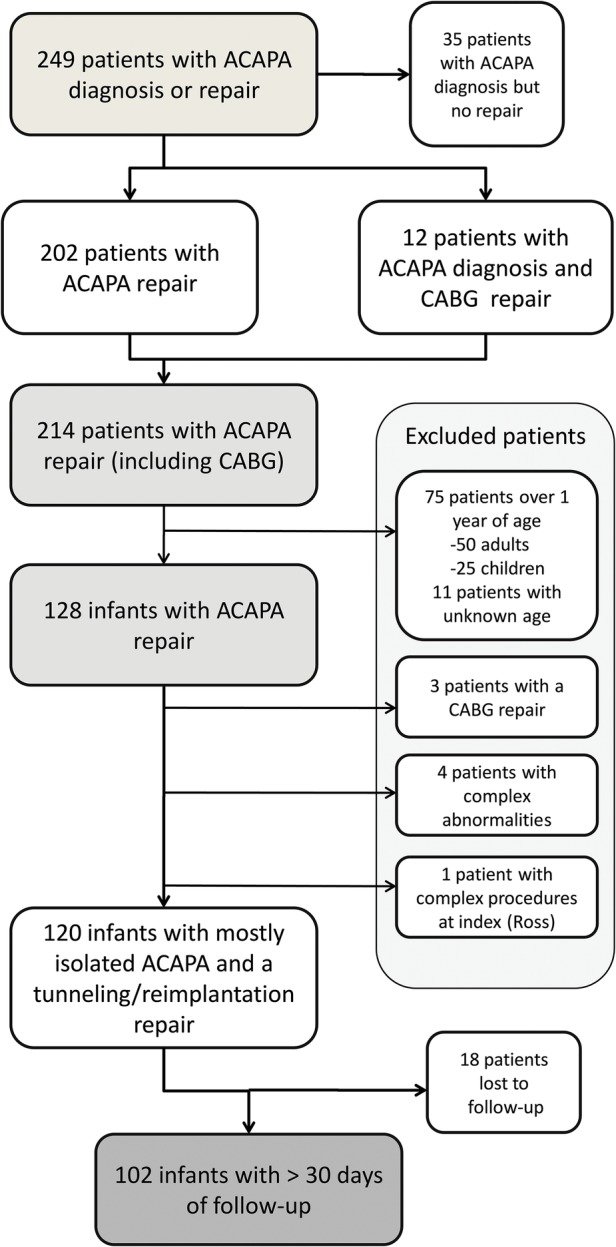
Patient inclusion and exclusion flow chart. ACAPA, anomalous origin of coronary artery from the pulmonary artery; CABG, coronary artery bypass grafting.

All data available on the patients undergoing a coronary artery procedure for a congenital cause between 2000 and 2013 were selected and anonymised. A total of 249 patients of all ages with either an ‘ACAPA diagnosis’ or an ‘ACAPA repair procedure’ were identified in the NICOR data set, which does not differentiate between the types of ACAPA repair procedures (ie, tunnelling or reimplantation). Out of these, 214 had an ACAPA repair, 128 of them being infants; 75 patients older than 1 year were excluded, as were 11 patients with an undocumented age, due to administrative errors. Since the objective of the study was to report the results in patients having ACAPA as their primary defect, we excluded four infants with complex abnormalities (one with partially anomalous pulmonary venous connections, one with atrioventricular septal defect and two with double-outlet right ventricle) and one patient with severe aortic valve disease requiring a Ross operation at index. We also excluded the three infants who were listed as having a coronary artery bypass grafting (CABG) procedure. Patient inclusion/exclusion details can be found in figure 1.

#### Statistical analysis

Frequencies are given as absolute numbers and percentages, continuous values as mean (SD) or median (IQR). Short-term mortality is reported based on 30-day life status (where follow-up is available). Comparisons of proportions were carried out with the Fisher Exact test. Estimates of long-term survival and freedom from reintervention were evaluated with the Kaplan-Meier method using mortality (all cause) and reoperations (coronary/mitral valve/pulmonary) as failure events. Owing to the low number of death events, we also used a competing risks method and this resulted in similar results to the Kaplan-Meier method.^7^ For brevity, only the results from the actuarial methods were reported. Predictor variables for short-term mortality were tested using the χ^2^ test. For long-term survival and freedom from reintervention we performed univariable analysis using the log-rank test and Cox proportional hazards regression (in the case of the mitral valve reintervention where more than 10 events were available). The low number of events did not permit a multivariable analysis, as to avoid over-parameterisation. Considered predictor variables for univariable analysis were: age (continuous), age (under and over 30 days), extracorporeal membrane oxygenation (ECMO), mitral valve disease and mitral valve procedure at index. Population characteristics were compared using the Mann-Whitney U test, t test and the χ^2^ test as appropriate. Statistical analyses were carried out with STATA/IC 11.2 (StataCorp LP, College Station, Texas, USA).

## Results

In total 120 infant patients with ACAPA and a coronary transfer or tunnel operation were identified, 102 (85%) having follow-up extending over 30 days. A detailed patient inclusion/exclusion flow chart can be seen in figure 1. Note that there were no early deaths in the group of patients excluded due to age (n=86), and two late deaths, comparable to the retained group. Short-term mortality/reintervention and long-term results data are calculated using the group of patients with >30 days of follow-up (n=102), while all results concerning the index procedure are calculated based on the whole group of 120 infants, as this data is complete and shows the status of each patient at the moment of the operation.

Patient demographic, clinical and procedure-related data are presented in table 1. Mitral valve regurgitation was the main associated pathology present at index or during follow-up in close to 20% of the patients. A summary of other miscellaneous associated malformations and procedures can be seen in table 2.

**Table 1:** Patient and procedure-related data in patients with ACAPA

*Demographic and follow-up data*	
Patients (n)	120
Age (days)	
Mean (SD)	133 (77)
Median (IQR)	119 (81–168)
Gender (n, %)	
Male	76 (63.3%)
Female	44 (36.7%)
Age group (n, %)	
Neonate (<30 days)	6 (5%)
Infant (1–12 months)	114 (95%)
Follow-up (y)	
Mean (SD)	4.7 (4.1)
Median (IQR)	3.7 (1.2–7.8)
*Clinical and procedure-related data*	
Patients (n)	120
Mitral regurgitation	24 (20%)
Other congenital defects	11 (9.2%)
MVR procedure at index	7 (5.8%)
MVR procedure after index	8 (6.6%)
ECMO	5 (4.2%)
Bypass time (min)	
Median (IQR)	113 (96–148)
Cross clamp time (min)	
Median (IQR)	55 (42–66)
Circulatory arrest during bypass, (n, %)	8 (6.6%)
Arrest time (min)	
Median (IQR)	4.5 (2–8.5)
Postop intubation time (hours)	
Median (IQR)	4.5 (2–8)
Hospital stay (days)	
Median (IQR)	17 (10–26)

ACAPA, anomalous origin of coronary artery from the pulmonary artery; ECMO, extracorporeal membrane oxygenation; MVR, mitral valve repair/replacement.

**Table 2:** Associated cardiac malformations and procedures at index in patients with ACAPA (not including mitral valve regurgitation and procedures), n=120

Malformations*		Procedures	
PDA	5	PDA closure	3
VSD	2	VSD closure	2
Pulmonary stenosis	1	Coarctation repair	1
Mitral valve stenosis	1	Pulmonary valve procedure	1
Coarctation of the aorta	1	Atrial procedure	1
Superior vena cava persisting to coronary sinus	1		

*Atrial septal defects (including patent foramen ovale) and patent arterial duct presence and correction are only reported to NICOR if considered clinically significant.

ACAPA, anomalous origin of coronary artery from the pulmonary artery; NICOR, National Institute for Cardiovascular Outcomes Research; PDA, patent arterial duct; VSD, ventricular septal defect.

A total of 13 centres performed ACAPA repair for infants in our time frame, with a patient volume median of 1.16 patients/year (ranging from a minimum of 0.08 patients/year to a maximum of 3 patients/year). No significant differences in outcomes were found between centres or based on patient volume.

### Short-term results

The short-term (30 day) mortality for patients with follow-up after discharge (n=102) was 1.9% (two patients died before 30 days), higher for neonates (16.7% vs 1%, p=0.1) and in patients who required postoperative ECMO after the repair (20% vs 1%, p=0.09). One patient was recorded with myocardial infarction (MI) at presentation and two had a postprocedural MI, although the criteria used for MI diagnosis and reporting to NICOR are not documented. One of these patients, a 4-month-old infant, suffered an MI early after the ACAPA repair. Shortly after he underwent a right outflow tract balloon dilation and was discharged alive after 30 days but lost to follow-up. The second patient, a 13 days old neonate, required ECMO after the ACAPA repair. She suffered an MI in the early postoperative period and underwent mitral valvuloplasty and an unknown coronary procedure at 20 days of age and died at 38 days of age. The other patient who died in our group, was a 6-month-old infant, with an associated patent ductus arteriosus that was ligated at the time of ACAPA repair. He died in the first postoperative day.

Five patients (4.2%) required ECMO postoperatively and one of them died early (described above), resulting in a 20% mortality for ECMO patients. Patients with mitral valve repair/replacement (MVR) at index did not have increased short-term mortality (0% with MVR vs 2.1% without MVR, p=0.8).

A total of nine (7.5%) patients required early reoperations. Out of these, four had unknown cardiac procedures, two had MVR, one had a right ventricular outflow tract dilation (described above), one had a MVR while one had a coronary reintervention and a mitral annuloplasty (being the only one who also died). All patients were under 6 months at the time of reoperation.

### Long-term results

**Figure 2:**
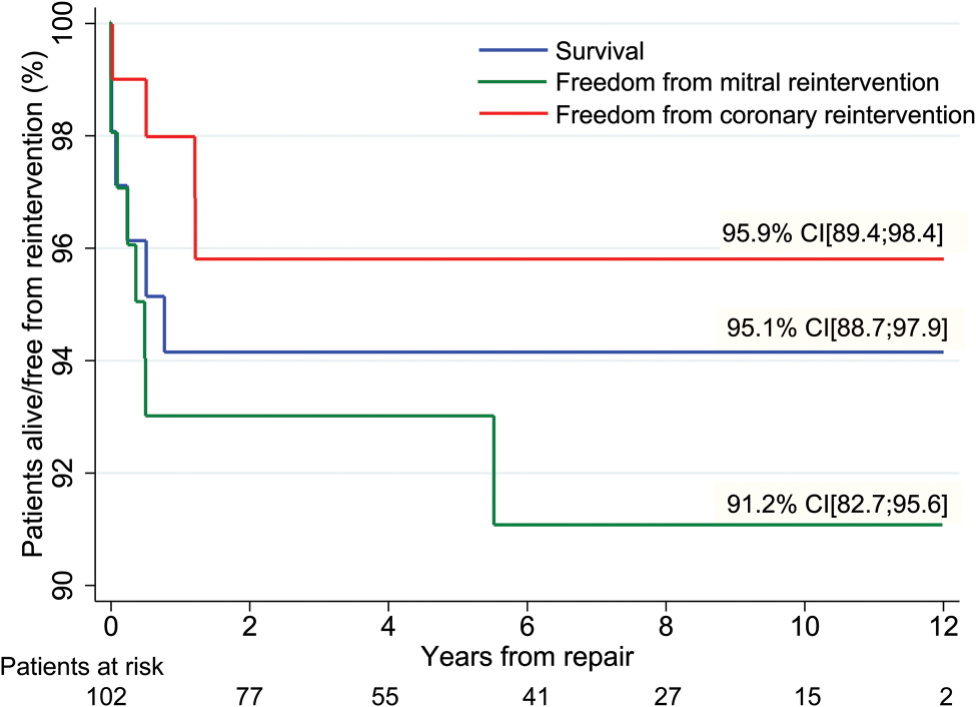
Long-term results estimates for patients with anomalous origin of coronary artery from the pulmonary artery, showing survival, freedom from coronary and mitral valve reintervention plots. Values shown are 10-year estimates, obtained using the Kaplan-Meier method. Vertical axis does not start at 0. Patients at risk are similar for all three survival functions.

**Figure 3:**
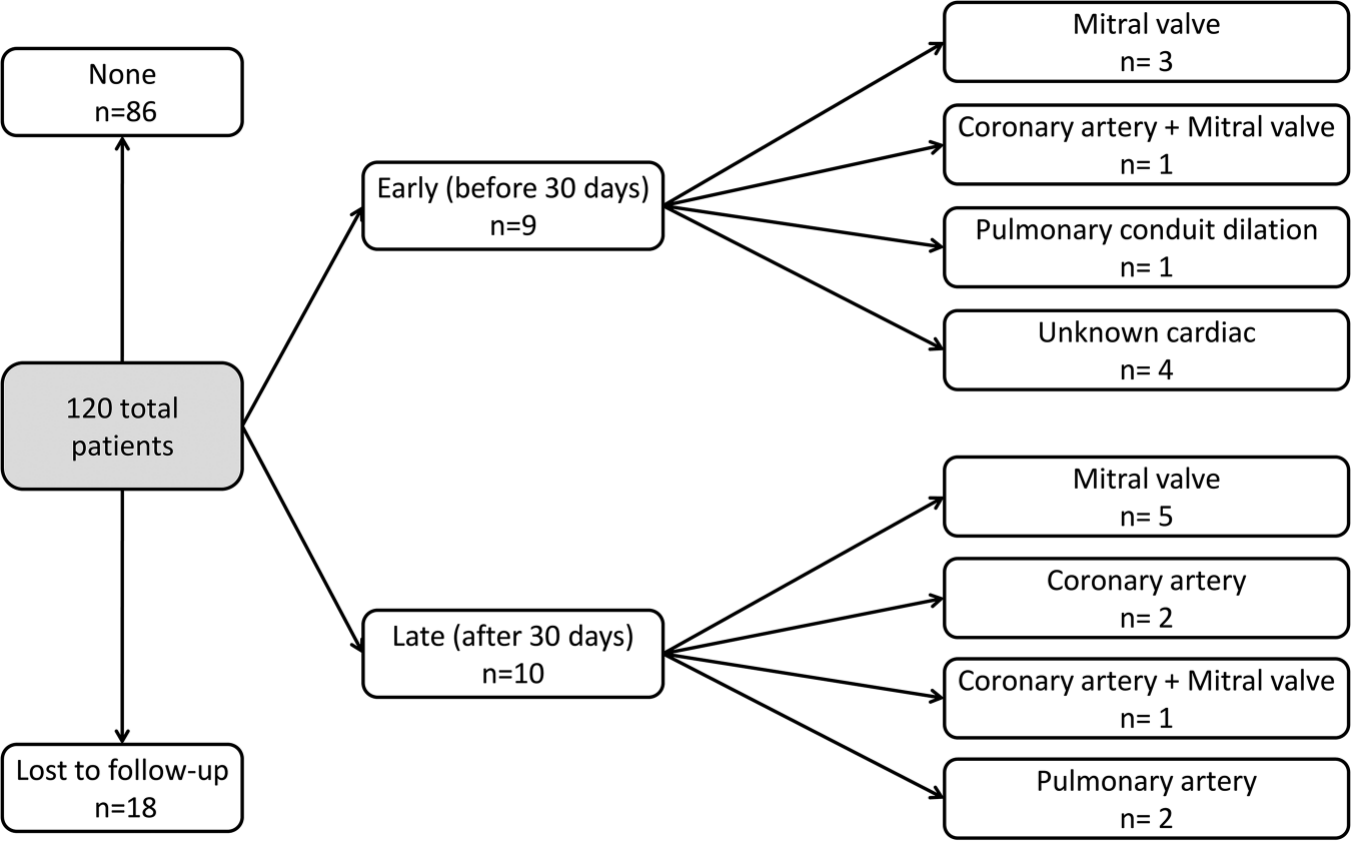
Flow chart showing reinterventions by type in patients with anomalous origin of coronary artery from the pulmonary artery patients, divided into early (before 30 days) and late (after 30 days). Some patients underwent both early and late reinterventions or more than one late reintervention.

Survival, freedom from coronary reintervention and freedom from mitral valve reintervention estimates at 12 years can be seen in figure 2. No patient died after 1 year of follow-up. The coronary artery reinterventions were performed in the first 2 years of follow-up (median 10.2 months), while the mitral valve reinterventions were performed in the first 5 years of follow-up (median 3.7 months). Three patients (2.9%) had pulmonary conduit and artery reinterventions: one early right ventricular outflow tract balloon dilation after 12 days, a balloon dilation of the pulmonary branches after 3 months and a pulmonary artery reconstruction after 1.8 years. Freedom from any reintervention (coronary, mitral or pulmonary) is 87.2% at 12 years, with all reinterventions (early and late) being summarised in figure 3.

The only risk factor identified for late mortality was the need for postoperative ECMO (p<0.001). Identified risk factors for coronary reintervention were age under 30 days (p=0.06) and the need for postoperative ECMO (p=0.02). In terms of mitral valve reintervention, the only identified risk factor was age under 30 days (p=0.002). The need for reinterventions did not adversely affect the long-term survival.

**Figure 4:**
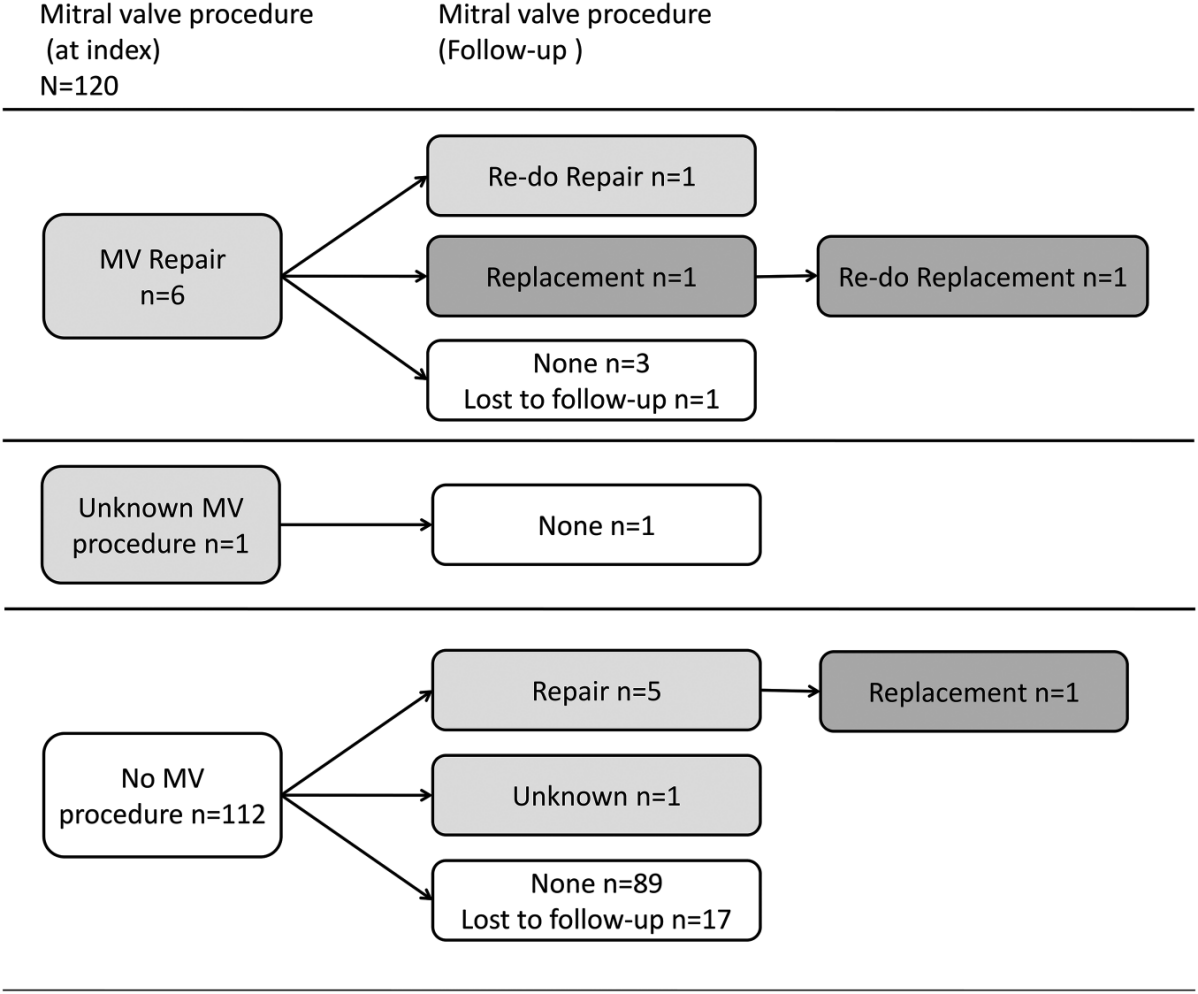
Flow chart showing mitral reinterventions by type in all patients. MV, mitral valve.

Of the four patients who required a coronary reintervention two also had a MVR at the same time, accounting for 2/8 of all patients with a mitral valve reintervention. Of the seven patients with a MVR procedure at index two (28.5%) also had a mitral valve reintervention. A detailed description of mitral valve procedures performed at index and during the follow-up can be seen in figure 4.

## Comment

This is, to the best of our knowledge by far the largest multicentre series presenting results of ACAPA correction in infants to date. Using information from a national audit database we report results from a national population that are both representative and generalisable, hence not limited to the results of experienced centres.

Similarly to previously published, smaller series,^8^ we decided to focus on infants, since this is a homogenous and distinct group. Having the least developed coronary collaterals, they require urgent treatment after diagnosis and present with various degrees of heart failure.

### Mortality risk factors

Early mortality after re-establishment of a dual coronary artery repair using coronary artery transfer or the tunnel operation (Takeuchi procedure) ranges between 0 and 23%.^2^ Direct coronary artery transfer is currently the most favoured technique after becoming popular through increasing experience with the arterial switch operation. The combined early mortality for patients having coronary transfer or Takeuchi in our series was 2.5%.

Use of postoperative ECMO was associated with increased early and late mortality in this study. This may be related to the critical condition at presentation in these patients which prompted postoperative support. In one of the largest series to date, patients who required postoperative ECMO were more likely to present with a low ejection fraction.^5^ Low left ventricle ejection fraction at presentation was found to be a risk factor for operative mortality in most series.^3^ ^4^ ^9^ ^10^ As stated above, we have not used this parameter in analysis due to limitations in the audit database. The fact that young age at operation has been associated with operative mortality, as in previously published results,^10^ further points to the conclusion that early presentation in neonates is often associated with critical heart failure.

Late deaths are rare after restoration of dual coronary system and ventricular recovery. In our study we had three deaths after 30 days, all before 1 year of follow-up, resulting in an actuarial survival of 94% at 10 years, which is comparable with previously published results.^3–5^

### Reinterventions

The largest series to date reported excellent freedom from reintervention rates in the range of 81–93% at 10 years.^3–5^ In our study, we also found freedom from all reinterventions (mitral, coronary and pulmonary) to be 87.2% at 12 years.

Age below 30 days was associated with an increased risk for coronary and mitral reintervention, while postoperative ECMO was linked to a higher risk of coronary reinterventions. Patients presenting in the neonatal period are more likely to have more severe ischaemia and mitral regurgitation because they are at the end of the spectrum of insufficient coronary collateralisation, as suggested by Azakie *et al*.^5^ This is a possible explanation for the higher risk of coronary and mitral reinterventions in this age group. The higher risk of mitral reintervention may also be related to the technical difficulties posed by repairing the mitral valve at this age.^2^ ECMO patients are more likely to have more severe ischaemia resulting in a poor ventricular function requiring postoperative support. This could explain the higher risk of coronary reintervention we identified in this subgroup.

In our group, two of four patients who required a coronary reintervention also underwent a MVR in the same operation, accounting for 2/8 of all patients who underwent MVRs in the follow-up. This naturally suggests that a failing coronary connection leads to aggravation of the mitral valve regurgitation. Since all coronary reinterventions in our study occurred during the first year of follow-up, a careful monitoring of myocardial ischaemia during this time interval is warranted.

The tunnel/intrapulmonary baffling procedure (Takeuchi operation) has its specific complications: supravalvular pulmonary stenosis and baffle leaks.^11^ In the present series we had two patients with late supravalvar pulmonary reinterventions and one patient who required an early balloon dilation of the pulmonary conduit.

### Limitations

Our study is derived from a national procedure audit database, thus shares all its limitations. The main downside of the NICOR database is its focus on procedural data, rather than patient-level data, since its primary objective is to allow for complex national quality index checks. This leads to a limited number of collected variables in order to ensure a high-quality index for the key procedure fields. Nevertheless, the NICOR curated database is sufficiently robust to allow fresh insights into both adult and congenital procedures.^12–14^ The main limitations in this study are the lack of clinical, operative and echocardiographic data. At the time of the analysis, CCAD did not differentiate between ALCAPA and ARCAPA, although considering the particularities of infants presenting with this anomaly, it is safe to assume that the majority are indeed patients with ALCAPA. In our own series of 26 ACAPA repairs performed in a single centre between 1982 and 2013, 23 patients had ALCAPA and only three had ARCAPA. Patients with ARCAPA presented in late adulthood, above the age of 46 years. In addition, we could not differentiate between the Takeuchi repair and coronary artery transfer in the national data set, so we were unable to compare the two procedures.

The lack of echocardiographic data did not allow an analysis of the LV performance and degree of mitral valve regurgitation following ALCAPA correction. This prevented us from drawing any conclusions with regards to the myocardial recovery and the fate of the mitral regurgitation after repair. A second study aimed at extracting clinical, operative and echocardiographic data is warranted to further characterise these groups.

Finally, we acknowledge the lack of long-term follow-up in 15% cases.

## Conclusions

These preliminary national results show that early outcomes are good and medium-term attrition acceptable. Poor outcomes are correlated with early presentation, being also linked to the need for postoperative circulatory support. Further clinical studies are needed to elucidate the best surgical approach in early childhood, the role and the timing of mitral surgery and the importance of myocardial perfusion tests in the early follow-up.
